# Determination of the PIC700 Ceramic’s Complex Piezo-Dielectric and Elastic Matrices from Manageable Aspect Ratio Resonators

**DOI:** 10.3390/ma14154076

**Published:** 2021-07-22

**Authors:** Lorena Pardo, Álvaro García, Franz Schubert, Antje Kynast, Timo Scholehwar, Alfredo Jacas, José F. Bartolomé

**Affiliations:** 1 Instituto de Ciencia de Materiales de Madrid, CSIC, C/Sor Juana Inés de la Cruz, 3, Cantoblanco, 28049 Madrid, Spain; alvarog@icmm.csic.es (Á.G.); ajacas@icmm.csic.es (A.J.); jbartolo@icmm.csic.es (J.F.B.); 2 PI Ceramic GmbH, D-07589 Lederhose, Germany; F.Schubert@piceramic.de (F.S.); a.kynast@piceramic.de (A.K.); t.scholehwar@piceramic.de (T.S.)

**Keywords:** bismuth sodium titanate, barium titanate, piezoelectricity, coupling, shear resonance, dielectric properties, elastic properties

## Abstract

Achieving good piezoelectric properties, such as the widely reported d_33_ charge coefficient, is a good starting point in establishing the potential applicability of piezoceramics. However, piezoceramics are only completely characterized by consistent piezoelectric-elastic-dielectric material coefficient matrices in complex form, i.e., including all losses. These matrices, which define the various alternative forms of the constitutive equations of piezoelectricity, are required for reliable virtual prototyping in the design of new devices. To meet this need, ten precise and accurate piezoelectric dielectric and elastic coefficients of the material, including all losses, must be determined for each alternative. Due to the difficulties arising from the coupling of modes when using the resonance method, this complete set of parameters is scarcely reported. Bi_0.5_Na_0.5_TiO_3_-based solid solutions are already commercially available in Europe and Japan. Here, we report a case study of the determination of these sets of material coefficients (*d_iα_*, *g_iα_*, *e_iα_* and *h_iα_*; *s^E^*^,*D*^*_αβ_* and *c^E^*^,*D*^*_αβ_*; *ε^T^_ik_* and *ε^S^_ik_*; and *β^T^_ik_* and *β^S^_ik_*), including all losses, of the commercial PIC700 eco-piezoceramic. Plate, disk, and cylinder ceramic resonators of a manageable aspect ratio were used to obtain all the material coefficients. The validation procedure of the matrices is also given by FEA modeling of the considered resonators.

## 1. Introduction

Piezoceramics of Bi_0.5_Na_0.5_TiO_3_-based solid solutions are already commercially available in Europe under the denomination, PIC700 [1]. Moreover, there are commercial ceramics based on the same material in Japan [2]. Achieving good piezoelectric properties, such as the widely reported d_33_ charge coefficient for sensors, is a good starting point in establishing their potential applicability. However, this knowledge is largely insufficient. Consistent piezoelectric-elastic-dielectric material coefficient matrices in complex form, i.e., including all losses, which define the various alternative forms of the constitutive equations of piezoelectricity, are required for reliable virtual prototyping in the design of new devices, which is a relatively inexpensive and time-saving procedure [3,4].

Finite Element Analysis (FEA) is widely used for device simulation. Nevertheless, its potential for modeling lead-free commercial piezoceramics is still insufficiently exploited. This is due to the lack of complete sets of data, including all losses, for many of these. The FEA modeling is often restricted to consider piezoceramics as lossless and even isotropic materials, which is just a rough approximation of their real performance. Piezoelectric ceramics have lossy properties, which are conveniently defined by complex values (P* = *P*′ − i*P*″) [5,6], and possess an induced anisotropy, created by the unique direction of the electric poling field. Piezoceramics are also dispersive and non-linear materials, and their set of properties only has validity in a given range of applied electrical and mechanical fields and frequencies.

The material parameter determination from complex impedance measurements at the electrically excited electromechanical resonances of piezoelectric ceramics and their analysis are strictly valid only for monomodal, uncoupled modes. Aiming to avoid mode coupling, the standard measurement guidelines on the resonance method [7,8] have established the resonator geometries to be used and their dimensional aspect ratios. High aspect ratios are recommended to separate the various modes of each resonator associated to each of its dimensions. It is recommended to use 5 resonators (2 bars, 2 plates, and 1 disc), whose characteristic resonance frequencies are in the interval from 100 kHz to a few MHz, for the full material characterization in the linear range. Standards were issued for the determination of the ten independent piezoelectric, elastic, and dielectric coefficients, which correspond to these 6 mm symmetry materials of each set of constitutive equations of the piezoelectricity in the reduced form.

The resonance method is widely spread due to its advantages [9], namely, easily available instrumentation, high repeatability, and high accuracy in terms of the results. However, it is commonly considered to lack consistency in the results obtained for the alternative forms of the sets of constitutive equations of piezoelectricity [7] that can be used in numerical modeling using Finite Element codes: These equations are as follows:

(1) $$T_{ij}=c_{ijkl}^{E}S_{kl}-e_{kij}E_{k}$$

(2) $$D_{i}=e_{ikl}S_{kl}-\varepsilon_{ij}^{S}E_{k}$$

(3) $$S_{ij}=s_{ijkl}^{E}T_{kl}+d_{kij}E_{k}$$

(4) $$D_{i}=d_{ikl}T_{kl}+\varepsilon_{ik}^{T}E_{k}$$

(5) $$S_{ij}=s_{ijkl}^{D}T_{kl}+g_{kij}D_{k}$$

(6) $$E_{i}=-g_{ikl}T_{kl}+\beta_{ik}^{T}E_{k}$$

(7) $$T_{ij}=c_{ijkl}^{D}S_{kl}-h_{kij}D_{k}$$

(8) $$E_{i}=-h_{ikl}S_{kl}+\beta_{ik}^{S}D_{k}$$

where *e_kij_*, *d_kij_*, *g_kij_*, and *h_kij_* are the piezoelectric coefficient matrices; *s^E^*^,*D*^*_ijkl_* and *c^E^*^,*D*^*_ijkl_* are the elastic compliances and elastic stiffness tensors, respectively, at a constant electric field (*E*), closed circuit, or constant electric displacement (*D*), open circuit; *ε^T^_ik_* and *ε^S^_ik_*, *β^T^_ik_*, and *β^S^_ik_* are the free (at constant, zero, stress (*T*)) and clamped (at constant, zero, strain (*S*)) relative dielectric permittivity matrices and dielectric impermeabilities or impermittivities (*β* = 1/*ε*), respectively. These equations are currently being simplified using Einstein´s summation convention of repeated subscripts (*i*, *k* = 1, 2, 3; *ij* = α; and *kl* = *β*, where α, *β* = 1, 2, …, 6) and the subscript 3 to denote the polarization direction in the material. The mentioned matrices’ inconsistency derives from the use of resonator modes at a relatively wide range of frequencies and whose microstructural characteristics or poling levels may not be identical. For this reason, it is a good practice to provide one or more validity and consistency criteria of the set of parameters determined [8].

To provide a better consistency between matrices, it was shown previously that all material coefficients can be determined from only 3 resonators and four modes of resonance [3,10]. Measurements of the radial and thickness extensional modes of thickness-poled thin disk can be used to obtain those parameters derived from the Length Thickness Extensional (LTE) mode of a bar and the Thickness Extensional (LTE) mode of a thin plate, which is thickness-poled. The full set of matrices is completed using well-known relationships between coefficients [3,10].

However, the thin plate for shear coefficient determination (e.g., *d*_15_, *s^E^*_55_, and *ε^S^*_11_) from the electrically-induced shear mode cannot be excluded from this reduced resonator set. Two types of difficulties arise when using the standard thin shear plate, which is length-poled and excited in thickness. They cause the associated parameters to be scarcely reported and affect the consistency of the matrices. On the one hand, longitudinal poling is a problem when dealing with materials of a high coercive field [11] or low dielectric breakdown [12]. On the other hand, the coupling of shear modes and other lateral and contour modes, which takes place in the shear resonance of standard length-poled shear plates, is unavoidable [3,4,13,14]. Unfortunately, this leads to the stringent requirement of aspect ratios between the length for poling (*L*), the width (*w*), and the thickness for electrical excitation (*t*) of the plate of *L*, *w* > 20 *t* [15] and to an underestimation of the piezoelectric coefficients [13].

To create an improved approach to the determination of the properties using the resonance method, we present, in this work, the determination of piezoceramic coefficients by the measurement of four resonance modes in three poled ceramic resonators, including a, non-standard, thickness-poled and shear plate [3]. An analysis of the complex impedance spectra was carried out using a widely tested iterative automatic method that overcomes the difficulties and limitations of the current standard procedures of measurement [7,8]. Noticeably, this method has been implemented in the analysis of all resonances under consideration [3,16,17,18]. Besides, the method is self-sustainable, as it does not need an initial estimate of the parameters. Some issues to consider when attempting to obtain weakly coupled resonances and reliable coefficients are discussed. Commercial PIC700 ceramics were studied, and the piezoelectric-dielectric-elastic matrices of material coefficients in complex form were determined for the alternative types of constitutive sets of piezoelectricity (Equations (1)–(8)). Besides, the validity of the matrices was tested by using them in Finite Element Analysis modeling for the reproduction of the experimental measurements.

## 2. Materials and Methods

### 2.1. Materials

Bi_0.5_Na_0.5_TiO_3_-based commercial lead-free (PIC700; PI Ceramic GmbH, Lederhose, Germany) poled ceramic resonators were studied in this work [19]. The ceramics were prepared from high-purity oxide precursors by the conventional ceramic method. Samples were cut and lapped to the desired dimensions. Afterwards, they were electroded and poled in an oil bath, according to the company standards [19], whose details are protected knowledge. All the resonators under study were poled to saturation in the same conditions to ensure the consistency of the results obtained from them. All measurements were taken for at least two samples—although statistical analysis is not an issue discussed in this work—to verify that the results are not affected by microstructure accidents. Changes from batch to batch could take place. The density of the considered samples was 5.49 g·cm^−3^.

### 2.2. Material Coefficient Determination

Piezoelectric, elastic, and dielectric complex coefficients in the linear range (*d_iα_*, *g_iα_*, *e_iα_*, and *h_iα_*; *s^E^*^,*D*^*_αβ_* and *c^E^*^,*D*^*_αβ_*; *ε^T^_ik_* and *ε^S^_ik_*; and *β^T^_ik_* and *β^S^_ik_*), accounting for all losses, were determined at room temperature from measurements of complex impedance curves. The data were automatically acquired from an HP-4192A LF precision impedance analyzer (Hewlett-Packard, Palo Alto, CA, USA) and controlled by a PC via a GPIB-PCIIA (National Instruments). An automatic iterative method explained elsewhere [3,16,17] was here used for the analysis of the curves for each resonance considered. Contrarily to standard methods, this method does not require additional measurements, besides those of the resonance. Four complex impedance spectra, Z*, of the electromechanical resonance modes of three poled ceramic resonators were analyzed. These modes are: (i) Shear mode of non-standard, thickness-poled, and longitudinally excited shear plates, (ii) Radial and (iii) Thickness extensional modes of thickness-poled thin disks; and (iv) Length extensional mode of a longitudinally poled cylinder. The software used is available to the scientific community [20].

**Figure 1:**
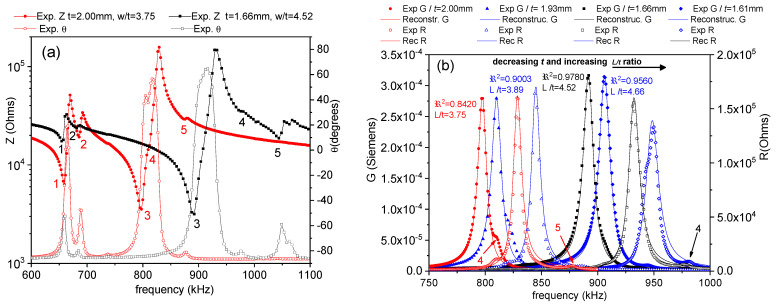
Shear resonance mode of a, non-standard, thickness-poled and longitudinally excited shear plate: (**a**) Impedance spectra in (*Z*, *θ*) plots, and (**b**) the same spectra in the equivalent (*R*, *G*) plots. In the (*Z*, *θ*) plots, the lines are only a “guide for the eye” in between the experimental points, and the frequency interval covers the lateral modes (marked as 1, 2, 4, and 5) at both sides of the fundamental shear mode (marked as 3). In the (*R*, *G*) plots, the symbols are the experimental data, and the lines are the reconstructed peaks after the calculation of the coefficients for the fundamental shear mode of the plates. The values of the regression factors for these calculations (*ℜ*^2^) are shown. Figure (**a**) shows two spectra: the one corresponding to the initial plate with *t* = 2 mm (red circles; full symbols for *Z* values and open symbols for *θ* values) and the one for the reduced plate with *t* = 1.66 mm (black squares; full symbols for *Z* values and open symbols for *θ* values). The other two dimensions, *L* = 7.49 mm and *w* = 7.45 mm, remain unaltered in the process. Figure (**b**) shows the two equivalent spectra for the same plates as in Figure (**a**), (red circles for *t* = 2 mm and black squares for *t* = 1.66 mm). In Figure (**b**) the full symbols are used for the *G* values, and open symbols are used for the *R* values. Additionally, in Figure (**b**) the spectra of an intermediate plate with *t* = 1.93 mm and *L*/*t* = 3.89 (blue triangles) and of a plate after a further thickness reduction to *t* = 1.61 mm and *L*/*t* = 4.66 (blue rhombus) are shown to complete the description of the resonance decoupling process.

Commonly, Z* is represented as the modulus (*Z*) and phase angle (*θ*) vs. frequency plots (Figure 1a). Here, we also use an equivalent representation: the peak of the real part of Z*, the resistance (*R*), and the peak of the conductance (*G*), the real part of the complex admittance, which is the inverse of the impedance (Y* = 1/Z*). This is a convenient plot for using the automatic iterative method (Figure 1b). After plotting these peaks, the frequencies of the maximum *G* and *R* values, *f_s_* and *f_p_*, respectively, are automatically determined by the software. For each of the considered modes, some material coefficients were directly determined by the automatic iterative solution of the impedance/admittance expression as a function of these coefficients and the resonator density (ρ) and dimensions (thickness between electrodes for electrical excitation (*t*); electroded surface area (*S*)) [3,16,17]. In each iteration of the analysis, the software solves a system of four equations for the values of Z* or Y* measured at four frequencies, including *f_s_* and *f_p_*, by a numerical method, until a convergence criterion of the last determined coefficients in comparison with the previous ones is met.

Besides, using well-known expressions among coefficients of the constitutive equations [3,10], several other coefficients related to the studied mode were also determined after the directly calculated ones. These are the electromechanical coupling factors (*k_x_*, where *x* = 33, 31, 15, *p* (radial mode of the disc) and *t* (thickness mode of the disc)) and the corresponding frequency numbers ($N_{x}=f_{s}^{x}\left(\mathrm{kHz}\right)\cdot D\left(mm\right)$, where $f_{s}^{x}$ is the series resonance of the x mode, and *D* is the leading dimension of the resonance), as well as the planar Poisson’s ratio.

After the directly obtained coefficients are available, a reconstruction of the spectra (Figure 1b) is carried out. Calculation of the complex impedance as a function of the frequency is conducted using the analytical expression that was just solved and the calculated coefficients. The residuals for these *R*- and *G*-reconstructed peaks (a residual being the difference between the experimental value for a given frequency and the value of the reconstructed peaks at the same frequency) give us a regression factor for the iterative analysis (*ℜ*^2^). This parameter accounts for the validity of the analytical expression and these coefficients for 1D modeling of this mode of resonance. The higher the coupling of the resonance with other undesired resonances, the lower the *ℜ*^2^. The closer the experimental resonance to a monomodal resonance, the closer *ℜ*^2^ is to 1.

### 2.3. Finite Element Modelling

The FEA modeling software (COMSOL Inc., Burlington, MA 01803, USA, COMSOL Multiphysics^®^ 4.3) was used. The piezoelectric device module (PZD) was used to simulate the piezoelectric response of the four resonance modes of the three resonators considered here. The mesh used typically has five nodes per wavelength, which allowed for simulating the modes of resonance that are excited, together with the fundamental resonance under study. The number of frequencies analyzed was chosen to obtain the needed resolution of each calculated spectra. The shear plate was simulated as a full 3D item. This typically results in a calculation time of up to 5 h for each sweep of 1000 frequencies. The cylinder and disk resonators were simulated using their rotational symmetry, which results in faster calculations. The 3D harmonic analysis used here provides the complex impedance values in a given interval of frequencies. The intervals of the frequency of interest in the present work were those of the experimental measurements.

## 3. Results and Discussion

### 3.1. Measurements and Calculation of the Piezoelectric, Elastic, and Dielectric Material Coefficients

#### 3.1.1. Shear Resonance of a, Non-Standard, Thickness-Poled and Longitudinally Excited Shear Plate

Thin PIC700 ceramic plates of a ratio between the length for electrical excitation to thickness for poling *L*/*t* = 3.75 were initially prepared with *t* = 2.00 mm. Figure 1a shows the experimental spectra in (*Z*, *θ*) plots for this resonator. In addition to the fundamental shear mode associated to the thickness of the plate, which takes place at *f_s_* = 797.7 kHz and is marked as 3, four other modes are measured. The resonances marked as 1 and 2 correspond to modes taking place at lower frequencies, which are associated with the larger lateral dimensions of the plate. Their overtones appear at periodically distributed frequencies. Those marked with 4 and 5 are coupled with the fundamental shear resonance, which is marked 3. This invalidates the sample for the accurate determination of complex material coefficients.

The major surfaces of the non-standard plate are not electroded for the electrical excitation of the resonance and the impedance measurement. It is therefore feasible to reduce the thickness, which increases the *L*/*t* ratio and the frequency of the fundamental resonance by polishing the major surfaces in steps of approximately 0.05 mm. Measurements were taken after each step for the control of the coupling. As the lateral dimensions remain unchanged, the frequencies marked 1 and 2 for the lateral modes are unaffected. The coupling diminishes as the shear frequency moves away from these frequencies to higher frequencies. Noticeably, these modes are weakened as their frequency is separated from the frequency for the fundamental shear. The calculations carried out in each step are not strictly valid for the accurate determination of the properties [21], but they allow for the quantitative evaluation of the coupling, using the regression factor for the iterative analysis (*ℜ*^2^). Figure 1a also shows the resonance spectra, measured again after the thickness of the plate was reduced in steps to a plate with *t* = 1.66 mm (*L*/*t* = 4.52), which has a virtually uncoupled fundamental shear mode taking place at *f_s_* = 892.0 kHz.

Figure 1b shows the alternative (*R*, *G*) plot for the two samples shown in Figure 1a and for the intermediate plate with *L*/*t* = 3.89. Both the experimental and reconstructed spectra, after calculation, are shown in Figure 1b, together with the increasing values of *ℜ*^2^, from the initial 0.8420 to the intermediate value of 0.9003 and up to 0.9780 for the sample of *t* = 1.66 mm. Figure 1b shows also the data for a plate with *L*/*t* = 3.89, after a further thickness reduction to *t* = 1.61 mm, which again shows a coupled shear mode due to interference, with modes 4 and 5 marked in the spectra of the *t* = 1.66 mm sample in Figure 1a. For this sample, *ℜ*^2^ is reduced to 0.9560. This indicates that the maximum decoupling of modes was surpassed, and the resonators evolve away from a monomodal resonance once again.

The described procedure does not follow the standard procedure for preventing coupling by the separation of frequencies of the resonance modes of the resonator, associated with each one of its dimensions, by measurement in high-aspect-ratio resonators. It is assumed that, in this way, the overtones of low-frequency modes become weaker at higher frequencies, and the same occurs in the case of coupling with the high frequency resonance of interest.

Nevertheless, previous work on thickness-poled shear plates has shown that undesirable overtones of lateral modes are activated by coupling when the shear mode frequency is in coincidence with their frequency. This occurs periodically. This periodical sequence of coupling–decoupling processes of the non-standard shear resonator was observed for a soft lead titanate-zirconate (PZT; four periods studied from *L*/*t* = 7.5 to 15) [3] and for a barium calcium titanate-zirconate (BCZT; five periods studied from *L*/*t* = 6 to 12) [19]. Besides, it was modeled by FEA for bismuth sodium barium titanate (BNBT; three periods modeled from *L*/*t* = 7.5 to 14.5) [22].

According to the results shown in Figure 1, the plate used here for the coefficient determination was the *t* = 1.66 mm one. This was efficiently decoupled from lateral modes, despite its low aspect ratio, compared with the standard recommended procedure. Besides, this was achieved using a method that is not feasible for the standard resonator, because it requires multiple steps for the removal of electrodes and re-electroding at room temperature.

The directly calculated coefficients in this mode are *ε^S^*_11_, *e*_15_, and *s^E^*_55_, and, from these, *ε^T^*_11_, *d*_15_, *h*_15_, *g*_15_, *s^D^*_55_, *c^E^*_55_, and *c^D^*_55_ are also calculated. Due to the symmetry *d*_24_ *= d*_15_, *e*_24_ *= e*_15_, *h*_24_ *= h*_15_, *g*_24_ *= g*_15_, *ε^T^*^,*S*^_22_ *= ε^T^*^,*S*^_11_, *s^D^*^,*E*^_44_ = *s^D^*^,*E*^_55_, and *c^D^*^,*E*^_44_ = *c^D^*^,*E*^_55_ are also determined. Additionally, the electromechanical coupling factor and frequency number, *k*_15_ and *N*_15_, are obtained from this resonance mode. These are shown in Table 1, Table 2 and Table 3.

#### 3.1.2. Resonances of a Thickness-Poled and Excited Thin Disk

(a) Extensional radial resonance of the disk

This is the only resonance mode for which it is necessary to conduct additional measurement outside the fundamental resonance spectrum to apply the iterative method. The series frequency of the first overtone of this mode (*f*_2*s*_) is used to calculate the first estimate of the planar Poissón´s ratio (*σ^P^*) from the ratio, *f*_2*s*_/*f_s_* [17].

**Figure 2:**
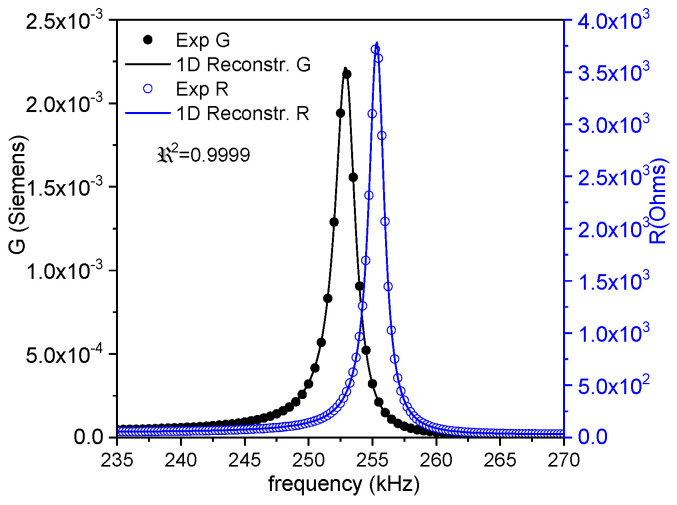
Fundamental radial extensional resonance mode of a thickness-poled and excited thin disk. Impedance spectra in an (*R*, *G*) plot of a disk with a diameter of 12 mm and *t =* 1.15 mm. The symbols are the experimental data, and the lines are the reconstructed peaks after the calculation of the coefficients. The value of the regression factor for this calculation (*ℜ*^2^) is shown. The black symbols and lines (*G* data) can be read in the left-y-axis, and the blue ones (*R* data) are in the right-y-axis.

Radial resonance-derived parameters are the most commonly reported, since it is the mode of the lower frequency of the disk, and it is not affected by coupling. Figure 2 shows the monomodal impedance spectrum of the radial mode as an (*R*, *G*) plot of a thickness-poled and excited thin disk with a diameter of 12 mm and *t =* 1.15 mm. The experimental and reconstructed spectra are shown. The coefficients from the iterative method lead to a a high regression factor *ℜ*^2^ = 0.9999.

The directly calculated coefficients in this mode are *ε^T^*_33_, *d*_31_, and *s^E^*_11_, which are the coefficients that can otherwise be obtained by the LTE of a thickness-poled bar. The coefficient *s^E^*_12_ is also determined in the radial mode from the Poisson´s ratio and given the expression *σ^P^* = (−*s*_12_*^E^*⁄*s*_11_*^E^*). Besides, *g*_31_, *s^D^*_11_, and *s^D^*_12_ are also calculated from the previous values, along with *s^D^*^,*E*^_66_ *= 2(s^D^*^,*E*^_11_ *− s^D^*^,*E*^_12_*)* and *c^D^*^,*E*^_66_ = 1/*s^D^*^,*E*^_66_. Due to the symmetry *d*_32_ *= d*_31,_ *g*_32_ *= g*_31,_ *s*_21_ *= s*_12_, and *s*_22_ *= s*_11_ are determined. Additionally, the electromechanical coupling factors and frequency numbers, *k_p_*, *N_p_*, and *k*_31_, were obtained. These are shown in Table 1, Table 2 and Table 3.

(b) Extensional thickness resonance of the disk

In comparison with the relatively easy to handle monomodal resonances of bars and plates, great care must be taken with the disc resonator and its dimensional ratio to avoid complex high-frequency resonance modes. These are the overtones of the planar mode or the contour modes, which are coupled with the high-frequency fundamental thickness mode of the disk. For this purpose, the recommended standard procedure is to have a diameter to thickness ratio (*D*/*t*) > 10. However, it is also widely reported that coupling takes place also under this condition [9,23,24,25].

**Figure 3:**
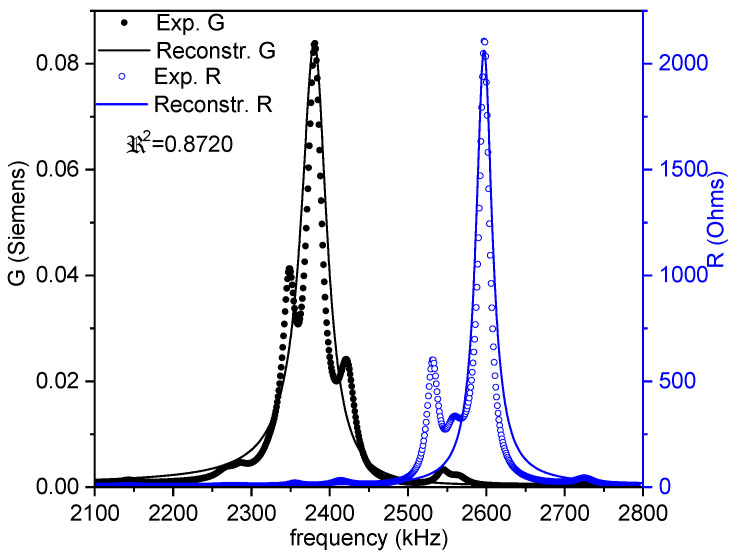
Fundamental thickness extensional resonance mode of a thin disk. The impedance spectra in an (*R*, *G*) plot of a disk with a 12 mm diameter and 1.00 mm thickness. The symbols are the experimental data, and the lines are the reconstructed peaks after the calculation of the coefficients. The value of the regression factor for this calculation (*ℜ^2^*) is shown. The black symbols and lines (*G* data) can be read in the left-y-axis, and the blue ones (*R* data) are in the right-y-axis.

Figure 3 shows the impedance curve in an (*R*, *G*) plot for the thickness extensional mode used for the evaluation of the parameters in this mode. A distorted spectrum is shown, which is the result of coupling. This deficiency would pose some problems with the accuracy of the material coefficients obtained, particularly the losses, as reflected by the regression factor of the calculation: *ℜ*^2^ = 0.8720. Undoubtedly, research must still be conducted with the aim of enhancing this experimental result, which compromises the overall determination of matrices. However, this type of spectra is what is commonly accepted as a manageable aspect ratio and impedance curve for analysis when working with the thickness modes of disks [9,23,24,25].

The directly calculated coefficients in this mode are *ε^S^*_33_, *h*_33_, and *c^D^*_33_. They can also be obtained from the LE mode of a thickness-poled thin plate. From these coefficients, *e*_33_ and *c^E^*_33_ are also calculated. At this stage of the process, the *ε^S^_ij_* matrix is complete, which allows for the calculation of the *β^S^_ij_* matrix. Additionally, the electromechanical coupling factor and frequency number, *k_t_* and *N_t_*, are obtained. These are shown in Table 1, Table 2 and Table 3.

#### 3.1.3. Longitudinal Resonance of a Longitudinally Poled and Excited Cylinder

**Figure 4:**
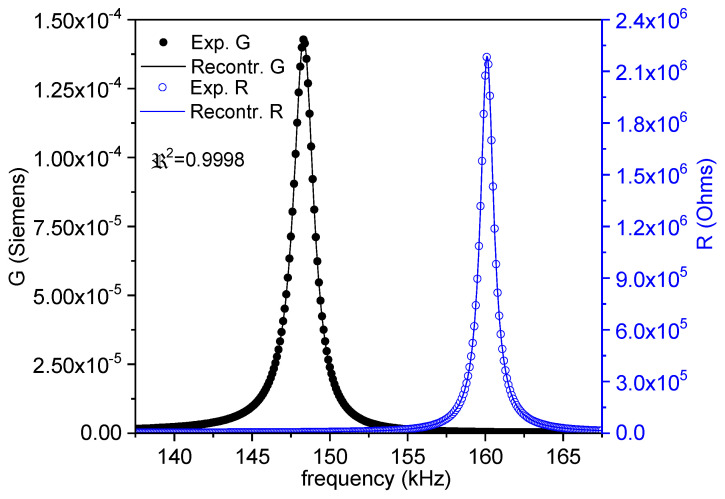
Fundamental length extensional resonance of a thickness-poled and excited cylinder. The impedance spectra in an (*R*, *G*) plot of a cylinder with a 6 mm diameter and 15 mm length. The symbols are the experimental data, and the lines are the reconstructed peaks after the calculation of the coefficients. The value of the regression factor for this calculation (*ℜ*^2^) is shown. The black symbols and lines (*G* data) can be read in the left-y-axis, and the blue ones (*R* data) are in the right-y-axis.

Figure 4 shows the impedance curve in an (*R*, *G*) plot for the length mode used for the evaluation of the parameters. As it corresponds to the mode of the resonator with the lowest frequency, this is uncoupled, and the regression factor from the calculation is high (*ℜ*^2^ = 0.9998). The directly calculated coefficients in this mode are *ε^T^*_33_, *g*_33_, and *s^D^*_33_. Due to the frequency dependence of the permittivity, the *ε^T^*_33_ determined in this mode is higher than the one determined in the thickness mode taking place at a higher frequency (Figure 3). Consequently, at this point, there is a need for an expert selection of the value that is more amenable for the validity of the matrices for FEA modelling [10].

At this stage of the process, with the coefficients determined for this resonance mode, the *g_iα_* and *ε^T^_ij_* matrices are complete, which allows the *β^T^_ij_* matrix to be calculated. From these coefficients, *d*_33,_ which completes the *d_iα_* matrix as well, and *s^E^*_33_ are also calculated. Due to the frequency dependence of the material coefficients, this *d*_33_ value at resonance is lower than the quasi-static one measured at 100 Hz. Additionally, the electromechanical coupling factor and frequency number, *k_t_* and *N_t_*, are obtained. These are shown in Table 1, Table 2 and Table 3.

#### 3.1.4. Combined Determination of the Remaining Material Coefficients

The combination of the coefficients obtained from the radial and thickness extensional resonances of a thin disk and the length extensional resonance of the cylinder, together with the well-known relationships among these coefficients [3,7,8,10], also gives the following:

(a) from the inversion of the matrices, we have the following expression:

(9) $$\;c_{33}^{E}=\frac{-s_{13}^{E}}{s_{33}^{E}(s_{11}^{E}+s_{12}^{E})-2s_{13}^{E}{}^{2}}\;$$

and, hence, we can obtain *s^E^*_13_. From *s^E^*_13_, the symmetry considerations provide *s^E^*_23_ *= s^E^*_13_, *s^E^*_31_ *= s^E^*_13_, and *s^E^*_32_ *= s^E^*_13_. This completes the *s^E^_αβ_* matrix and allows the calculation of its inverse, *c^E^_αβ_*, which at this point was only lacking the values of *c^E^*_11,_ *c^E^*_12_, and *c^E^*_13_, to be completed. This also ensures consistency between the stiffness and compliance matrices.

(b) knowing *c^E^*_11,_ *c^E^*_12_, and *c^E^*_13_, we can make use of Equation (62) in [8] to obtain *e*_31_ from the following:

(10) $$e_{31}=d_{31}\left(\;c_{11}^{E}+c_{12}^{E}\;\right)+d_{33}c_{13}^{E}\;$$

This completes the *e_iα_* matrix.

(c) by the relationships between the coefficients, we can calculate *h*_31_ using the following expression:

(11) $$h_{31}=\frac{e_{31}}{\varepsilon_{33}^{S}}$$

This completes the *h_iα_* matrix.

(d) making use of Equation (29) in [8], we can obtain *s^D^*_13_ from the following:

(12) $$s_{13}^{D}=s_{13}^{E}-\frac{d_{31}d_{33}}{\varepsilon_{33}^{T}}$$

and, similarly, by the symmetry considerations, we can also determine *s^D^*_23_ *= s^D^*_13_, *s^D^*_31_ *= s^D^*_13_, and *s^D^*_32_ *= s^D^*_13_. This completes the *s^D^_αβ_* matrix and allows the calculation of its inverse, *c^D^_αβ_*, which at this point was lacking the values of *c^D^*_11_*, c^D^*_12_, and *c^D^*_13_, to be completed. This completes all the matrix determinations, as shown in Table 1, Table 2 and Table 3, and determines the consistency between the *c^D^_αβ_* and *s^D^_αβ_* matrices.

The PIC700 eco-piezoceramic has been considered a good replacement for PIC 255 (PI Ceramic GmbH, Lederhose, Germany) material [1]. PIC255 is a soft-type modified lead zirconate-titanate, which is characterized by a moderate permittivity, high thickness coupling factor, high charge coefficient, and low mechanical quality factor (ratio *P*′/*P*″ for elastic coefficients) [19]. Applications of this ceramic type are in low-power ultrasonic transducers, non-resonant broadband systems, and force and acoustic sensors. PIC700 is additionally characterized by a lower planar coupling factor, higher electromechanical anisotropy [26], and lower density (Table 3).

When standard procedures are used, this is not a sufficiently precise method for determining a physically meaningful and mathematically coherent set of properties. To obtain a coherent set of matrices suitable for FEA analysis, these results must be optimized, using a suitable algorithm to minimize the overall error.

Here, we have used a validity check at each considered resonance mode through the reconstruction of the measured spectrum and considering only results with high enough *ℜ*^2^ values. Additionally, we have used a, non-standard, thickness-poled and longitudinally excited shear plate producing accurate complex shear coefficients, as the shear mode can be efficiently decoupled from other modes. The procedure reported here for the calculation of the coefficients by combining parameters that were obtained from previously analyzed spectra is not a universal solution, as there are options for calculating the indirectly determined coefficients [8,10].

### 3.2. Validation of the Piezoelectric, Elastic, and Dielectric Material Coefficients

The validity of the coefficients shown in Table 1, Table 2 and Table 3 will be evaluated in the following.

#### 3.2.1. Meaningful Losses

Holland’s criteria [26] for the material to be passive, in the sense of not being an energy generator, are given for the matrices of complex coefficients by the following expressions:

(13) $$\left|s^{E\prime \prime }{}_{11}\right|\geq \left|s^{E\prime \prime }{}_{12}\right|$$

(14) $$(s^{E\prime \prime }{}_{11}\cdot s^{E\prime \prime }{}_{33})\geq {\left(s^{E\prime \prime }{}_{13}\right)}^{2}$$

(15) $$(s^{E\prime \prime }{}_{11}\cdot \varepsilon^{T\prime \prime }{}_{33})\geq {\left(d^{\prime \prime }{}_{31}\right)}^{2}$$

(16) $$(s^{E\prime \prime }{}_{33}\cdot \varepsilon^{T\prime \prime }{}_{33})\geq {\left(d^{\prime \prime }{}_{33}\right)}^{2}$$

(17) $$(s^{E\prime \prime }{}_{44}\cdot \varepsilon^{T\prime \prime }{}_{11})\geq {\left(d^{\prime \prime }{}_{24}\right)}^{2}\;\mathrm{or},\;\mathrm{equivalently},$$

(18) $$(s^{E\prime \prime }{}_{55}\cdot \varepsilon^{T\prime \prime }{}_{11})\geq {\left(d^{\prime \prime }{}_{15}\right)}^{2}$$

(19) $$s^{E\prime \prime }{}_{33}\cdot (s^{E\prime \prime }{}_{11}+s^{E\prime \prime }{}_{12})\geq 2{\left(s^{E\prime \prime }{}_{13}\right)}^{2}$$

(20) $$\varepsilon^{T\prime \prime }{}_{33}\cdot (s^{E\prime \prime }{}_{11}+s^{E\prime \prime }{}_{12})\geq 2{\left(d^{\prime \prime }{}_{31}\right)}^{2}$$

These criteria were checked, and they are fulfilled by the set of data in Table 1, Table 2 and Table 3, which provide the first consistency criteria of the accomplished material coefficients.

#### 3.2.2. Finite Element Analysis

**Figure 5:**
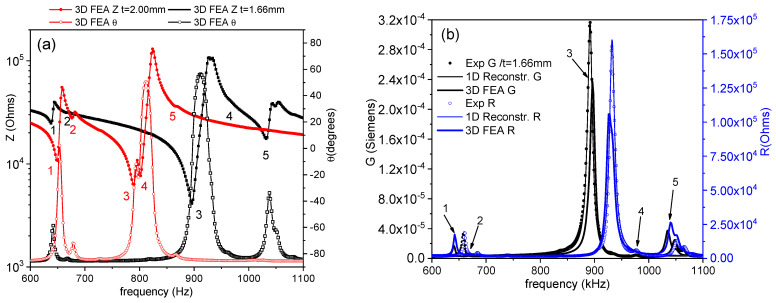
3D FEA modeled shear resonance of a, non-standard, thickness-poled and longitudinally excited shear plate (*L*): (**a**) impedance spectra in (*Z*, *θ*) plots and (**b**) spectra in the equivalent (*R*, *G*) plots. In the (*Z*, *θ*) plots, the lines are only a “guide for the eye” in between the modeled points. In Figure (**a**), the spectra for plates with *L* = 7.49 mm, w= 7.45 mm, and with an initial *t* = 2 mm (red circles) and reduced *t* = 1.66 mm (black squares) are shown. Full symbols are used for the *Z* values and open symbols for the *θ* values. The corresponding experimental (*Z*, *θ*) plots are given in Figure 1a. In the equivalent (*R*, *G*) plots in Figure (**b**), the 3D modeled spectrum of the plate with a reduced *t* = 1.66 mm is shown as a black thick line for the *G* peak and blue thick line for the *R* peak. For comparison, the experimental spectrum and the 1D modeled one (the spectra reconstructed using the iterative method) are also shown in Figure (**b**). The symbols are the experimental data of *G* (closed black circles) and *R* (open blue circles), and the thin lines are the reconstructed peaks (1D model) (black line for *G* and blue line for *R*). In Figure (**a**,**b**), the frequency interval covers the lateral modes (marked 1, 2, 4 and 5) on both sides of the fundamental shear mode (marked as 3).

Figure 5a shows the FEA modelled (/*Z*/, *θ*) spectra, including the lateral and the fundamental shear modes, for the coupled and decoupled resonances of the non-standard shear plates of a thickness *t* = 2.00 mm and *t* = 1.66 mm, respectively, whose experimental spectra are shown in Figure 1a. Both were calculated using the values of the complex coefficients shown in Table 1, Table 2 and Table 3. Figure 5b shows the (*R*, *G*) spectra for the plate used for the material coefficient determination (*t* = 1.66 mm). The height and frequency, where the main resonances are positioned in the FEA modeled spectra, are in good agreement with their respective experimental data, marked as 3 for the fundamental shear mode. It is worth noting that the agreement is also good for the secondary modes, marked as 1, 2, 4, and 5. The calculated parameters show the validity of the reproduction of all details of the spectra.

**Figure 6:**
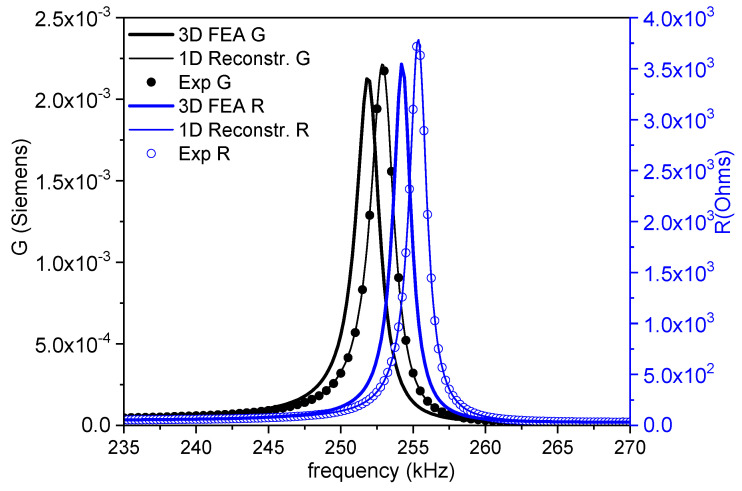
Fundamental radial extensional resonance mode of a thickness-poled and excited thin disk. The FEA-modeled (thick lines) impedance spectrum in an (*R*, *G*) plot of a disk with a diameter of 12 mm and a thickness of 1.15 mm. The experimental data (symbols) and the reconstructed spectrum after the calculation of the parameters in a 1D model (thin lines) are also shown for comparison. Black symbols and lines (G data) can be read in the left-y-axis, and the blue ones (*R* data) are in the right-y-axis.

**Figure 7:**
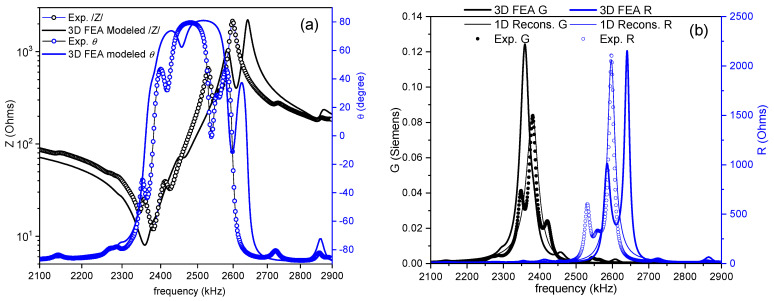
Fundamental thickness extensional resonance of a thickness-poled and excited thin disk. 3D FEA-modeled impedance spectrum: (**a**) (*Z*, *θ*) plot; and (**b**) equivalent (*R*, *G*) plot of a disk with a diameter of 12 mm and a thickness of 1.00 mm, shown as thick lines. The two types of plot also show the experimental spectrum (symbols). Figure (**b**), (*R*, *G*) plots showing the reconstructed spectrum after the calculation of the parameters (1D model) as thin lines. Black symbols and lines (*Z* and *G* data) can be read in the left-y-axis, and blue ones (*θ* and *R* data) are in the right-y-axis.

**Figure 8:**
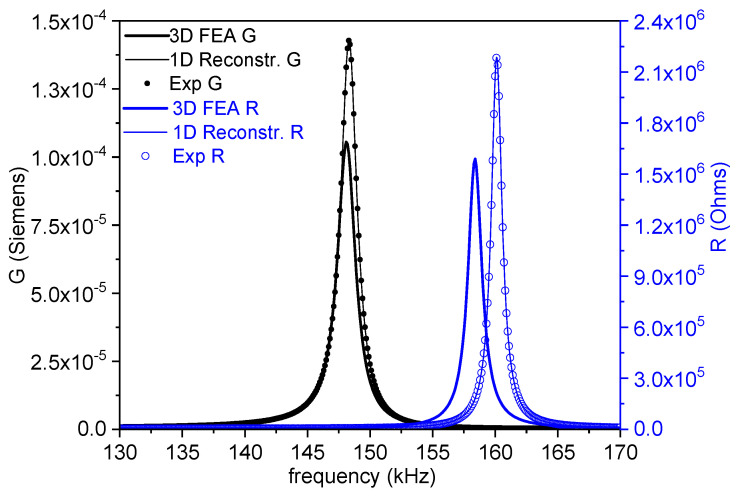
Fundamental length extensional resonance of a thickness-poled and excited cylinder. 3D FEA-modeled impedance spectrum in an (*R*, *G*) plot of a cylinder with a diameter of 6 mm and a length of 15 mm (shown as thick lines). The experimental and reconstructed spectra after the calculation of the parameters (1D model) are also shown as symbols and thin lines, respectively, for comparison. Black symbols and lines (*G* data) can be read in the left-y-axis, and blue ones (*R* data) are in the right-y-axis.

Figure 6, Figure 7 and Figure 8 show the FEA modeled spectra in (*R*, *G*) plots in the fundamental radial and thickness extensional modes of the thin disk and length extensional mode of the cylinder. The monomodal resonances are well modeled (Figure 6 and Figure 8).

Figure 7 shows a good reproduction of the positions for the minimum and maximum impedance modulus (Figure 7a) and the maxima of the main *R* and *G* peaks (Figure 7b) of the coupled resonance. Besides, all secondary features of the experimental spectrum in the distorted thickness extensional mode are also reproduced in the FEA-modeled curves.

The overall comparison between experimental and 3D FEA modeled spectra shown in Figure 5, Figure 6, Figure 7 and Figure 8 is a positive criterion for the validity of the complex material coefficients shown in Table 1, Table 2 and Table 3 in a frequency interval from 100 kHz to at least 3 MHz.

## 4. Conclusions

A non-standard strategy for obtaining an uncoupled shear resonance mode of a thickness-poled and longitudinally excited shear plate of PIC700 eco-piezoceramic was described here. It allows for the use of an analytical expression of the resonance, which is strictly valid for a monomodal resonance, on samples with an aspect ratio as low as *L*/*t* = 4.52, whereas the standard measurement procedure requires aspect ratios higher than 20. This strategy led to an accurate determination of all related shear material complex coefficients from a manageable aspect ratio plate, along with the material losses.

All piezo-dielectric and elastic matrices of the PIC700 piezoceramic for the alternative forms of the sets of constitutive equations of piezoelectricity were obtained from additional measurements of the disks and cylinders. An analysis of the impedance curves was made by the automatic iterative method. A quantitative evaluation of the validity of the complex coefficients, obtained at each resonance using the regression factor of the recalculated spectra (*ℜ*^2^), was accomplished. The selection of spectra with a high *ℜ*^2^ allows for the use of resonators of a manageable aspect ratio. The matrices of the coefficients are consistent and show the validity of PIC700 material modeling using an FEA in a frequency interval from 100 kHz to a few MHz.

## Figures and Tables

**Table 1:** Complex elastic coefficients of the PIC700 eco-piezoceramic ^(^*^)^.

***s^E^*^,*D*^** ***_αβ_*** ***= s*** **′ *+ is*″/** **10^−12^·** **m^2^** **·** **N^−1^**	***s^E^*_11_**	***s^E^*_12_**	***s^E^*_13_**	***s^E^*_33_**	***s^E^*_55_**	***s^E^*_66_**	***s^D^*_11_**	***s^D^*_12_**	***s^D^*_13_**	***s^D^*_33_**	***s^D^*_55_**	***s^D^*_66_**
*s* **′**	**8.5998**	**−2.0200**	1.673	8.9803	**20.7702**	21.24	8.5219	−2.0979	2.004	7.4639	18.6114	21.24
*s* **″**	**−0.0663i**	**+0.0154i**	−0.012i	−0.114i	**−0.2550i**	−0.163i	−0.0577i	+0.024i	−0.03i	−0.0486i	−0.2322i	−0.163i
***c^E^*^,*D*^** ***_αβ_*** ***= c*** **′ *+ ic*″/** **10^10^ N** **·** **m^−2^**	***c^E^*_11_**	***c^E^*_12_**	***c^E^*_13_**	***c^E^*_33_**	***c^E^*_55_**	***c^E^*_66_**	***c^D^*_11_**	***c^D^*_12_**	***c^D^*_13_**	***c^D^*_33_**	***c^D^*_55_**	***c^D^*_66_**
*c* **′**	13.102	3.686	−3.127	12.299	4.8139	4.708	14.056	4.641	−5.019	**16.092**	5.3722	4.708
*c* **″**	+0.107i	+0.034i	−0.044i	−0.164i	+0.0591i	+0036i	+0.043i	−0.029i	+0.049i	**+0.033i**	+0.067i	+0036i

^(^*^)^ Directly calculated parameters are quoted in bold.

**Table 2:** Complex piezoelectric coefficients of the PIC700 eco-piezoceramic ^(^*^)^.

***d_iα_ = d*** **′ + *id*** **″** **/** **10^−12^** **C·N^−1^**	***d*_31_**	***d*_33_**	***d*_15_**	***e_iα_ = e*** **′ + *ie*** **″** **/** **C·m^−2^**	***e*_31_**	***e*_33_**	***e*_15_**
*d*′	**−21.1408**	89.685 (**)	102.9606	*e*′	−6.357	11.0862	**4.9590**
*d*″	**+1.4046i**	−3.162i	−4.4218i	*e*″	+0.265i	+0.5252i	**−0.1520i**
***h_iα_ = h*** **′ + *ih*** **″** **/** **10^8^ V·m^−2^**	***h*_31_**	***h*_33_**	***h*_15_**	***g_iα_ = g*** **′ + *ig*** **″** **/** **mV·N^−1^**	***g*_31_**	***g*_33_**	***g*_15_**
*h*′	−15.173	**26.4709**	11.2442	*g*′	−3.6952	**15.632**	20.9383
*h*″	−0.408i	**+0.5634i**	+0.5044i	*g*″	+0.1393i	**−0.0180i**	+0.6777i

^(^*^)^ Directly calculated parameters are quoted in bold. (**) The quasi-static value is 120 × 10^−12^ C.N^−1^ at a Berlincourt d(sub 33)-meter (100 Hz).

**Table 3:** Complex dielectric coefficients, Poisson´s ratios, electromechanical coupling factors, and frequency numbers of the considered resonances of the PIC700 eco-piezoceramic ^(^*^)^.

***ε^S,T^_ik_ = ε*** **′ + *iε*** **″** **/*ε*_0_**	***ε*^S^_11_**	***ε*^S^_33_**	***ε^T^*_11_**	***ε^T^*_33_**	***β^S,T^_ik_ = β*** **′+*iβ*** **″** **/10^−4^/*ε*_0_**	***β^S^*_11_**	***β^S^*_33_**	***β^T^*_11_**	***β^T^*_33_**	***Poisson´s Ratio (σ^P^)***
*ε*′	**554.02**	**472.31**	496.43	**648.00**	*β*′	17.948	21.073	20.029	15.414	0.235
*ε*″	**−41.78i**	**−32.46i**	−37.54i	**−22.1i**	*β*″	+1.353i	+1.448i	+1.515i	+0.526i	+0.00002i
***kx* = *k*** **′ + *ik*** **″**	***k*** **_31_**	***k*** **_33_**	***k*** **_15_**	***k_p_***	***k_t_***	***N*** ***_x_*** **/kHz·mm**	***N*** **_33_**	***N*** **_15_**	***N_p_***	***N*** ***_t_***
*k*′	0.07632	0.41102	0.32239	0.14737	0.43482		2231.47	1480.72	3021.04	2381.00
*k*″	−0.00405i	−0.00504i	−0.00028i	−0.00783i	−0.00776i					

^(^*^)^ Directly calculated parameters are quoted in bold.

## Data Availability

The data presented in this study are available on request from the corresponding author.
